# Lung pneumonia severity scoring in chest X-ray images using transformers

**DOI:** 10.1007/s11517-024-03066-3

**Published:** 2024-04-09

**Authors:** Bouthaina Slika, Fadi Dornaika, Hamid Merdji, Karim Hammoudi

**Affiliations:** 1 University of the Basque Country UPV/EHU, San Sebastian, Spain; 2 Lebanese International University, Beirut, Lebanon; 3 Beirut International University, Beirut, Lebanon; 4 IKERBASQUE, Basque Foundation for Science, Bilbao, Spain; 5 INSERM, UMR 1260, Regenerative Nanomedicine (RNM), CRBS, University of Strasbourg, Strasbourg, France; 6 Hôpital Universitaire de Strasbourg, Strasbourg, France; 7 Université de Haute-Alsace IRIMAS, Mulhouse, France; 8 University of Strasbourg, Strasbourg, France

**Keywords:** Automatic prediction, Chest X-ray, Severity quantification, Vision transformer

## Abstract

**Abstract:**

To create robust and adaptable methods for lung pneumonia diagnosis and the assessment of its severity using chest X-rays (CXR), access to well-curated, extensive datasets is crucial. Many current severity quantification approaches require resource-intensive training for optimal results. Healthcare practitioners require efficient computational tools to swiftly identify COVID-19 cases and predict the severity of the condition. In this research, we introduce a novel image augmentation scheme as well as a neural network model founded on Vision Transformers (ViT) with a small number of trainable parameters for quantifying COVID-19 severity and other lung diseases. Our method, named Vision Transformer Regressor Infection Prediction (ViTReg-IP), leverages a ViT architecture and a regression head. To assess the model’s adaptability, we evaluate its performance on diverse chest radiograph datasets from various open sources. We conduct a comparative analysis against several competing deep learning methods. Our results achieved a minimum Mean Absolute Error (MAE) of 0.569 and 0.512 and a maximum Pearson Correlation Coefficient (PC) of 0.923 and 0.855 for the geographic extent score and the lung opacity score, respectively, when the CXRs from the RALO dataset were used in training. The experimental results reveal that our model delivers exceptional performance in severity quantification while maintaining robust generalizability, all with relatively modest computational requirements. The source codes used in our work are publicly available at https://github.com/bouthainas/ViTReg-IP.

**Graphical abstract:**

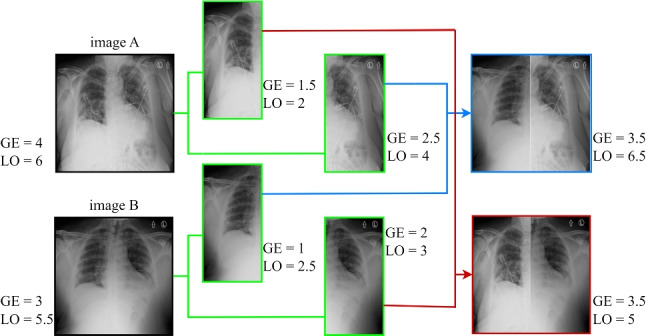

## Introduction

The number of deaths caused by coronavirus disease-19 (COVID-19) continues to rise even after vaccination by mandatory policies in most countries [1, 2]. Many physicians have turned to new tactics and technologies due to the increased impact of the pandemic on healthcare systems around the world. Chest radiographs (CXR) offer a relatively noninvasive method to track disease progression [2, 3]. CXR imaging is becoming more popular and more widely used worldwide, as demonstrated by many recent studies [4–9]. Since the diagnosis relies on the detection of imaging features and observation of their course and spread over the period of the disease start, CXR imaging devices are more widely accessible than CT scanners, which are more affordable. [10]. In addition, because portable CXR units are available, imaging can be performed within a stationary unit, which significantly reduces the risk of contamination transmission[6, 7, 11]. Finally, CXR imaging in patients with respiratory complaints is considered a commonly accepted practice in medicine [12] and it has been shown to provide insightful information about disease progression [9]. Numerous studies have examined CXR images, particularly those of patients with COVID-19 [4, 8, 13, 14], having bilateral anomalies, and ground-glass opacity in the interstitial space. Determining the severity of a patient’s disease is an important help of CXR assessment by physicians to guide disease management. As a result, several current studies have concentrated on severity grading to quantify the severity of lung disease [5, 9]. Disease severity can help physicians to determine the appropriate treatment and monitoring for each patient. Radiology services often employ experienced physicians for whom determining the severity of a CXR is not an easy task. The use of a computer to assist in clinical diagnosis could simplify this challenging work for medical professionals. In this research, we developed and examined a model that can predict lung pneumonia severity based on CXR and can be used to support patient care management. Escalation or de-escalation of care, particularly in the intensive care unit (ICU), may be based on the capability to assess the severity of pulmonary infection. Over time, a patient’s response to treatment and disease progression can be objectively and quantitatively tracked using an automated method. We anticipate that the usage of CXRs from a global pool of patients with pulmonary infections and normal patients can direct to a reliable and generic computer-aided severity grading of lungs. Throughout the study, we are interested in investigating the performance of our proposed model in predicting a scalar representing severity, rather than just classifying images as infected or uninfected. Recent work has shown that Deep Learning can be employed to solve regression cases such as estimating the age of faces [15], predicting the beauty of faces [16], and evaluating the risk score of breast cancer disease progression [17].

Our task is a regression task where we need ground-truth scores for supervised learning. Specifically, in this study, we develop, train, and validate a transformer-based deep neural network qualified for achieving the required score prediction. Multiple scoring systems can be applied using the CXRs of both infected and normal patients. In this way, we can evaluate the feasibility of computerized assessment of the severity of the lung towards assistance to support precise diagnosis and therapy. Although transformer-based architectures have been widely used recently [18], most research focuses on solving a classification problem rather than a regression problem as in the case of our research. We made our source codes accessible to the general public in order to entice further scholars to utilize them as a standard for their research: https://github.com/bouthainas/ViTReg-IP.

Below is a summary of our significant contributions:

- Formulation of a generalized and outperforming method based on a vision transformer (ViT) to expect the severity of a lung with infection.
- Derivation of mixing and fusing data augmentation methods, originally developed for classification tasks, as a scoring augmentation stage for our regression solution to generate a larger dataset.
- Carrying out a comparative study by exploiting *state-of-the-art* databases (RALO, Brixia, Danilov et al. COVID-19 and Cohen COVID-19) and eight different deep learning models (COVID-NET, COVID-NET-S, ResNet50, InceptionNet, XceptionNet, Swin Transformer, MobileNetV3, and Stonybrook Feature Extraction) and conducting a set of ablation studies showing the relative contribution of separate parameters in our ViTReg-IP.

The remainder of the paper is organized as follows: Related studies and a review of the *state-of-the-art* are described in Section 2. The description of our proposed generalized pneumonia severity quantification model is presented in Section 3. The performance evaluation, including the datasets used and the experimental results, as well as a detailed evaluation of the performance of each approach in severity assessment, are presented in Section 4. In Section 5, we interpret and discuss the obtained results. Section 6 summarizes the results and provides some concluding notes.

## Related work

COVID-19 has monopolized the focus and economic resources of investigators in several fields such as digital technologies, artificial intelligence, and data science from the start of the pandemic [19, 20]. Shi et al. [21] and Islam et al. [22] state that there are several techniques based on artificial intelligence that can be implied in medical image analysis for COVID. The authors categorized earlier work based on various tasks, including radiological feature extraction, disease diagnosis, image segmentation, process for noninvasive imaging, and severity quantification. At the very beginning of the outbreak, Oh et al. [23] proposed training convolutional neural networks (CNN) to analyze CXRs for hypothetical early diagnosis and thus better treatment of patients based on symptoms of pneumonia. Researching deep learning techniques was also investigated in [24] for autonomous assessment of CXR images to provide healthcare with accurate tools for COVID-19 screening and patient diagnosis. Furthermore, Sunnetci et al. [25] utilized chest X-ray images and introduced a method employing six classifiers. In two training phases, the top five classifiers were selected, and features were extracted using the Bag of Features method. The prediction of the class employed Majority Voting. The growing accessibility of COVID-19 patient CXR datasets during the outbreak has focused a lot of investigation exertions on diagnosis-oriented image interpretation investigations. There are so many studies that apply AI techniques to the acquisition, segmentation, and classification of imaging data for COVID-19, whether using CXRs or CT scans, that it would be difficult to cover them all [21, 26–28]. We refer only to those related to our work and the most recent emergent challenges.

Even though the CXR imaging modality is frequently used in multiple healthcare facilities, AI-driven solutions have been proposed for supervision and pneumonia severity inspection for COVID-19, especially those that predict a score, as is done in our study. The first paper was by Irmak et al. [29] and was based on a quantitative CXR assessment [30]. However, this study needs readers with more expertise to confirm the consistency of the severity score. After that, the COVID-Gram [31] and the deep learning applied outcomes of Liang et al. [32] were used for COVID-19 identification based on CXR abnormality. The degree of lung pneumonia was determined in the work of Colombi et al. [33] to indicate the disease severity. Another remarkable work was COVID-NET-S [34], one of the earliest research projects on COVID-19 severity estimation, in which the authors developed a deep neural network to forecast extent scores based on CXR images. To do this, they had to train their model on a huge dataset. Several features from a neural network already trained on CXR datasets other than COVID-19 are considered for their predictive score on the estimate of COVID-19 severity ratings in [35]. Ridley created a unique type of deep learning network called the Convolutional Siamese Neural Network (CSNN) to produce a score called pulmonary X-ray severity (PXS) for COVID-19 patients that was well correlated with radiologist assessments [36]. In [37], a transfer learning method is applied from a large dataset to a small one to show a clear relationship between a lung severity score rating and automated model prediction. Improved generalizability was attained in a subsequent study by the same authors [38]. In [39], pneumonia localization networks were used to produce a geographic extent severity score that was annotated and linked with experts’ evaluations on 94 CXRs in addition to lung segmentation models. In [40], the authors predicted two scores for quantifying lung severity. The authors in [34] used an architecture for COVID-19 detection and Monte Carlo cross-validation. These were performed on 396 CXRs, measuring the relationship with respect to expert annotations. CheXNet, which was trained to predict COVID-19 severity using a unique dataset, was proposed in the study by Kwon et al. [41]. An end-to-end deep learning model was used in [42] to predict a multi-regional score; Brixia score based on CXR images (CXR), indicating the severity of lung damage in COVID-19 patients. This architecture used a large dataset and needed several pieces of training for segmentation and subsequent prediction.

**Fig. 1:**
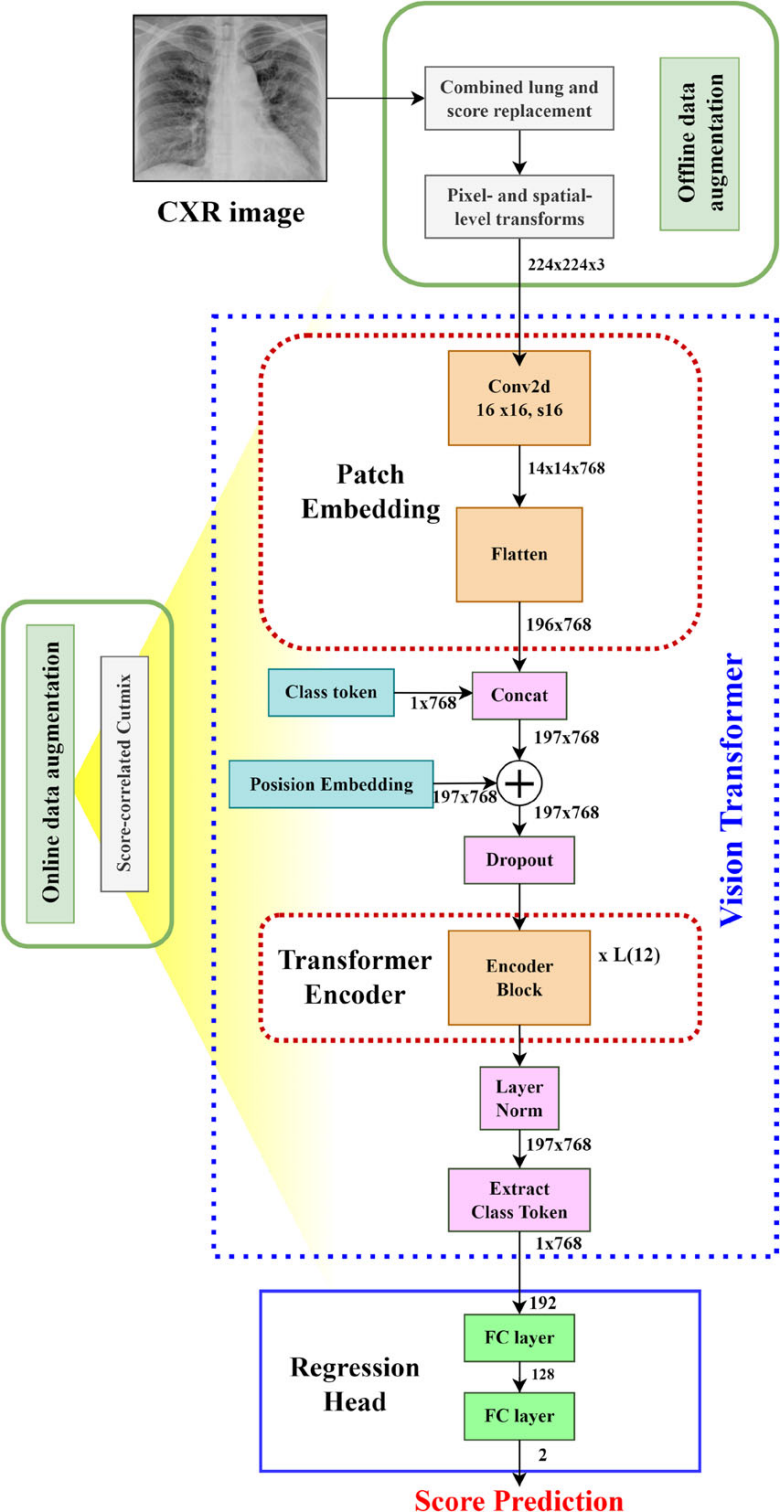
Overview of the proposed ViTReg-IP model

When using sophisticated architectures to analyze datasets with thousands of images, the computational overhead associated with such approaches can become excessive. Furthermore, focusing exclusively on COVID-19 images within datasets while neglecting other types of pneumonia may lead to suboptimal results. These preliminary investigations demonstrate the feasibility of evaluating CXR images and emphasize the need for technological solutions to meet the requirements of this visually complicated task. Furthermore, it is clear that small labeled datasets need to be processed and that models that are computationally affordable are needed to provide meaningful and explanatory results. In overcoming these challenges, it becomes clear how important it is to use a model with modest training parameters and to employ augmentation methods.

In contrast to the above strategies, our research shows how a specialized, straightforward technological tool designed to organize and manage severity scores can achieve high performance and robustness while keeping computational costs to a minimum.

**Fig. 2:**
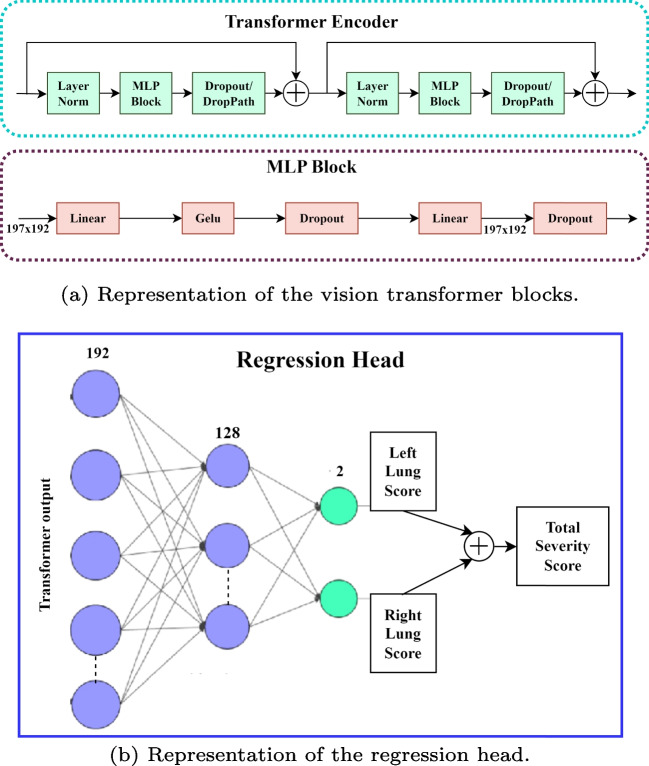
Detailed representation of the feature extraction blocks and the regression block in the proposed method

## Proposed methodology

### Combined feature extraction models

Given a CXR image with dimensions $H\times W\times C$, our goal is to predict the respective lung infection severity score. $H\times W$ is the spatial resolution of the input image and *C* is the number of channels. In our approach, a regressor is backed by the vision transformer ViT [43]. Figure 1 depicts the schematic diagram of our proposed model ViTReg-IP.

#### Vision transformer backbone

In this study, the ViTReg-IP model is founded on the vision transformer [43]. The deep neural network has already been pre-trained on ImageNet [44] in order to initialize the parameters. The non-hierarchical ViT design of the deep neural network was used in this study to underpin the architecture of the proposed model used to evaluate the functionality of the computational severity of lung disease. The ViT reshapes the input CXR image into a series of flattened 2D patches, where each patch has size $P$ $\times$ $P$ and $N = \frac{H\times W}{P^2}$ is the number of image patches. Using a trainable linear projection, we translate the vectorized image patches ${\textbf {x}}_p \in \mathbb {R} ^{P^2 \times C }$ into a *D*-dimensional embedding space. We learn certain position embeddings that are added to the patch embeddings to obtain the position information that encodes the spatial patch information. Thus, the encoding of the *N* patches is represented by the $N \times D$ matrix ${\textbf {z}}_0$ as follows:

1 $$\begin{aligned} {\textbf {z}}_0 = [{\textbf {x}}^1_p {\textbf {E}}; {\textbf {x}}^2_p {\textbf {E}};...; {\textbf {x}}^N_p {\textbf {E}}] + {\textbf {E}}_{pos}, \end{aligned}$$

where ${\textbf {E}} \in \mathbb {R} ^{P^2 \times C \times D }$ is the patch embedding projection, and ${\textbf {E}}_{pos}$ is the position embedding [43]. The Multihead Self-Attention (MSA) and Multi-Layer Perceptron (MLP) blocks are included in all *L* layers of the transformer encoder (2) and (3). Consequently, the following can be expressed as the output of the $l^{th}$ layer:

2 $$\begin{aligned} {{\textbf {z}}'_{l} = MSA (LN({\textbf {z}}_{l-1}))+{\textbf {z}}_{l-1}}, \end{aligned}$$

3 $$\begin{aligned} {\textbf {z}}_{l} = MLP(LN({\textbf {z}}'_{l}))+{\textbf {z}}'_{l}, \end{aligned}$$

where *LN*(. ) represents the layer normalization operator and ${\textbf {z}}_{l}$ is the encoded image representation at layer *l*. The configuration of a transformer encoder is illustrated in Fig. 2a. In our tests, we use a tiny ViT backbone with $(W, H) = (224,224)$, $C = 3$, $P = 16$, $L = 12$, and $D = 192$ [43].

#### Regression head

There are two Fully Connected (FC) layers in the regression head. It accepts the CLS token supplied by the last layer of the ViT backbone as input. Given that the infection levels in the two lungs are independent of one another, it then computes two predictions (for the left and right lungs) for the score. Thus, the ViT’s classification layer is substituted with two new fully connected layers that make up the regression head. Two linear layers, one with 128 neurons and the other with 2 neurons, make up this system. An illustration of the regression head is shown in Fig. 2b. To determine the extent of infection, individual score acts as a prediction for the severity of the left lung and the right lung. The network’s final output is the predicted score, which is the sum of the two output scores and ranges in value from 0 to 8.

4 $$\begin{aligned} {\textbf {p}} = [p_l; p_r ] = FC_2(FC_1({\textbf {CLS}}_{\textbf {L}})), \end{aligned}$$

where $FC_1$ and $FC_2$ are the two trainable fully connected layers respectively, ${\textbf {CLS}}_{\textbf {L}}$ is the ${\textbf {CLS}}$ token extracted from the final layer of the transformer, and ${\textbf {p}}$ is the predicted vector including the left and right lung scores. The final global output score is the sum of these two scores, $p= p_l +p_r$.

The final prediction of our VitReg-IP model, *p*, corresponds to the predicted CXR severity. Using a specific loss function, the whole network is trained by comparing this value to the ground-truth score, which is the actual score obtained through radiological labeling. It is important to note that although our proposed model produces predicted scores for each lung, the training data do not necessarily need to include ground-truth scores for each lung because the loss function is dependent on the global score of the whole lung.

### Data augmentation

All CXR images employed in this study underwent data normalization, a crucial phase that guarantees that each input parameter pixel has a uniform data distribution. This accelerates convergence during the training of the model. Moreover, to facilitate the act of training the deep neural networks, all CXR images were reformatted to identical dimensions of size $224 \times 224 \times 3$. We construct our deep neural network by applying successive operations in order to convert the CXR input data into the projected severity scores (e.g., geographic extent score, lung opacity score, Brixia score, and COVID score). The efficiency and effectiveness of our network are highly dependent on the accessibility to data as well as the preparation of training and test data.

If learned weights perform well in the training set but poorly in the test set, these models are overfitted. In the context of this study, we need to extend the size of the dataset used to avoid overfitting, which prevents the generalization of the model. Indeed, in precision health, there is often a lack of input data due to the novelty of the tackled topics and the high cost of labeling by medical experts [45]. In our architecture, the size of CXR images is increased by operating dropping and merging data augmentation methods.

More specifically, the data augmentation in this study involves the creation of new training images obtained from the original CXR training data using the combined offline lung and score replacement (inspired by the lung replacement method [46]) and the online score-correlated CutMix derived from the simple CutMix [47]. These two augmentation methods increase the data variety and potential of deep neural networks in terms of robustness and accuracy. The above two augmentation methods were developed and adapted for our regression problem and thus are used to generate the augmented images as well as their corresponding ground-truth scores which are the geographic extent (GE) and the lung opacity (LO) [48]. The geographic extent represents the area of infection of the lung infected and the lung opacity reflects the degree of opaqueness of this infection viewed on the CXR image.

#### Combined lung and score replacement

**Fig. 3:**
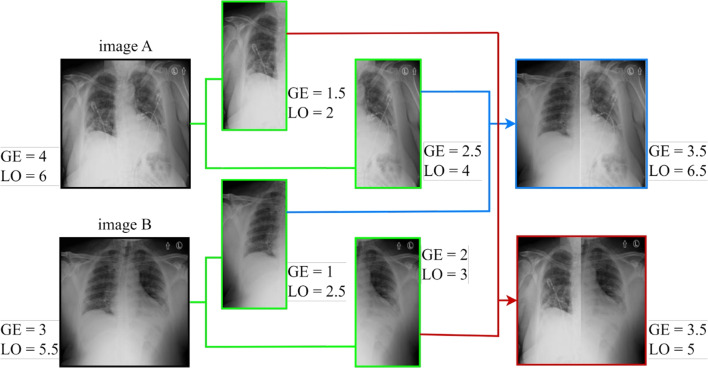
Combined lung and score replacement method applied on CXR images

We involved a lung replacement procedure previously proposed for a classification problem but in an improved version for the case of regression. The principle is based on replacing the left or right lung of a given patient with the opposite lung from a CXR of another patient. Lung replacement was applied to CXRs of the same class in [46] to increase the training data. However, since we have a regression problem in our case, we can use the lung replacement for any two CXR images. Additionally, the left and right lung scores of the original images’ left and right halves are added to determine the new ground-truth scores of the two consequent images. Thus, we replace both the lung and the score. In order to determine the ground-truth scores of the produced images, the individual lung actual scores are joined along with the blended lung parts. Individual ground-truth scores must be provided in order to apply this strategy. An illustration of the process is shown in Fig. 3. We used this technique on the training set of the RALO dataset [40], which initially contained 1878 photos. A combined lung and score replacement was applied to these images, resulting in two sets of synthetic images since we have two types of severity scores. The new training dataset now consists of a total of 5634 CXR images.

**Fig. 4:**
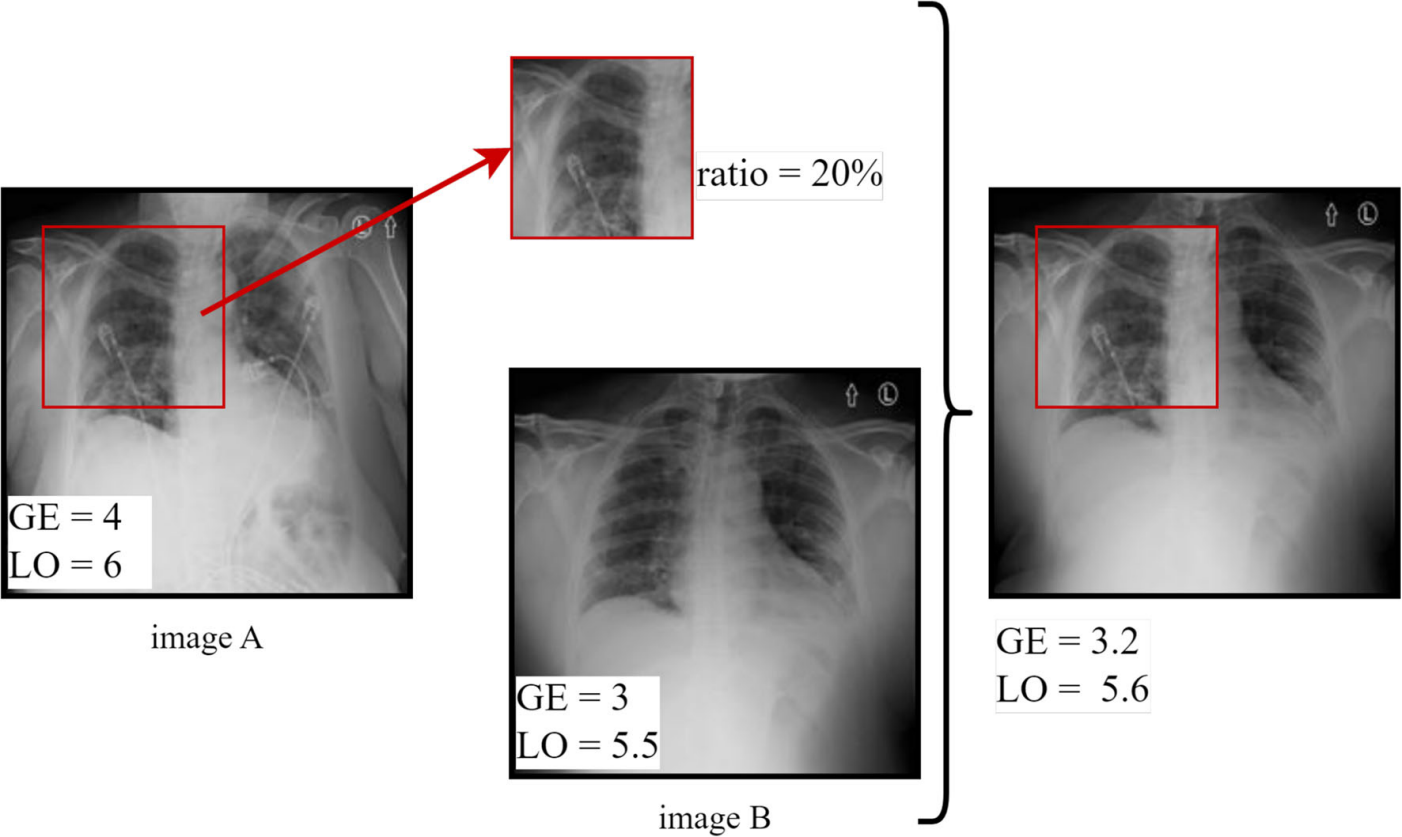
Score-correlated CutMix method applied on a CXR image

#### Score-correlated CutMix

The CutMix technique produces a locally realistic image by switching out a single image region for a patch from another training image. Figure 4 illustrates how we use CutMix to create a new image by replacing a part of image *A* that has been cropped with another image *B* whose size is randomly selected within a specified range. This technique, where one image is CutMixed with other images from the same batch at each epoch, was employed during online training. Our goal is to compute the updated ground-truth score of the synthesized image because our work involves a regression problem. As a result, we develop a score-correlated CutMix by applying the traditional CutMix (which is entirely image-based) to our regression problem. The ground-truth labels are calculated using a weighted average of the ground-truth scores of the two images, taking into account the total amount of pixels in the merged images, as shown in (5).

5 $$\begin{aligned} \overline{y} = \lambda *y_A + (1-\lambda )*y_B, \end{aligned}$$

where $\lambda$ is the ratio between the size of the substituted area and the total size of the image, $y_A$ and $y_B$ are the ground-truth scores of images *A* and *B*, respectively, and $\overline{y}$ is the new ground-truth score [47].

**Table 1:** Summary of severity labeled CXR datasets used in our study

Dataset	Data size	Annotations	Score range
RALO[34]	2373	GE, LO	[0-8]
Brixia[49]	4695	Brixia	[0-18]
Danilov et al. COVID-19[50]	1364	COVID score	[0-6]
Cohen COVID-19[51]	192+94	Brixia score+(GE, LO)	[0-18]+[0-8]

Efficient deep network training requires a significant amount of data. From a few images, it is possible to generate many more augmented images using the combined lung and score replacement method. Additionally, using the score-correlated CutMix during model training enables the creation of diverse images and their corresponding relative scores.

### Loss function and optimizer

$L_1$ loss, also known as Absolute Error Loss, is the loss function chosen for training. The used loss function is described in (6).

6 $$\begin{aligned} \mathcal{{L}} = \sum _{i=1}^{N_b} |p_{i}-\hat{p}_{i}|, \end{aligned}$$

where ${p}_i$ and $\hat{p}_i$ are the ground-truth score and the predicted score of the $i^{th}$ image, respectively. $N_b$ is the size of the batch. The experimental results revealed that when trained on the modified RALO dataset of CXR data using *L*1 Loss, ViTReg-IP had the best performance in comparison to other loss functions.

The stochastic gradient descent (SGD) optimizer is employed to change the model’s parameters. Its role is to modify model weights in order to reduce the loss function. This optimizer is chosen after testing varied optimizers and comparing the model’s performance.

## Performance evaluation

### Datasets

The main goal of this study is to examine the feasibility of deep learning-based computer assistance in assessing the severity of lung disease. To this end, we evaluate our solution as well as other deep neural networks capable of assessing CXR images of patients with different severities of lung infections. To this end, several CXR datasets were used in this study [34, 49–51]. Table 1 summarizes the datasets used.

#### RALO dataset

In our study, we employed the Radiographic Assessment of Lung Opacity Score (RALO) dataset [34]. The RALO dataset was recorded and graded by Stony Brook Medicine to offer researchers a defined COVID-19 dataset. Two renowned radiologists assessed the dataset, which consists of 2373 CXRs, to perform an additional COVID-19 severity analysis. The RALO dataset is divided into 1878 training images and 495 test images. The two evaluation criteria utilized in the radiological assessment are the geographic extent (GE) and the lung opacity (LO). The right and left lungs are evaluated separately, and the geographic extent of lung involvement caused by morning opacification is rated as follows: 0 = no engagement; 1 = 25%; 2 = 25%-50%; 3 = 50%-75%; and 4 means more than 75% involvement. After adding the scores, the total score for geographic extent (right + left lung) ranges from 0 to 8. The opacity level was scored for the right and left lungs individually. It ranges from 0 through 8. The 0 represents no opacity, 1 for ground-glass opacity, 2 for a mixture of consolidation (less than 50%) and ground-glass opacity, 3 for a mixture of consolidation (more than 50%) and ground-glass opacity, and 4 for complete opacification. The total score for the extent of opacity, obtained by adding the scores for the right and left lungs, ranges from 0 to 8 points [40].

#### Brixia dataset

The Brixia dataset, compiled from a dataset of 4695 CXR images matching the number of images acquired for patient monitoring in ICUs during the pandemic, was one of three datasets used to perform our tests [49]. The following annotations describe the relative Brixia score. The lungs are split into six zones, three for each lung when viewed from the anteroposterior (AP) or posteroanterior (PA) angle. Depending on the type and severity of lung abnormalities, a score of 0 (no abnormalities), 1 (interstitial infiltrates), 2 (interstitial and alveolar infiltrates, interstitial dominance), or 3 (interstitial and alveolar infiltrates, alveolar dominance) is assigned for each region. The six scores can be combined to get a Global Score ranging from 0 to 18.

#### Cohen COVID-19 dataset

The dataset by Cohen et al. COVID-19 is also used [51]. This collection consists of CXR images collected from numerous locations around the world, at different resolutions, and various image quality factors. Two subsets of this dataset were exploited in our analysis:

- The CXR labeled with the Brixia scores subset is used. A qualified staff member and a trainee radiologist with 22 and 2 years of experience, respectively, prepared the corresponding Brixia scores for the CXR in this subgroup. The collected dataset consists of 192 CXRs that were fully annotated using the Brixia scoring system.
- We also used a set of 94 CXR images from the COVID-19 imaging dataset, which is available to the general public. Physicians for each patient indicated that they were all COVID-19 positive. Ratings of geographic extent and lung opacity are used to label these images.

#### Danilov et al. COVID-19 dataset

In this dataset, the authors provide a collection of CXR images from positive and negative COVID-19 patients. There are a total of 1364 images. Of these, 580 images show COVID-19 positive results (43%), while 784 images show no results at all (57%). Each image was assigned a score between 0 and 6, with 0 representing no abnormalities and 6 representing a severe case of COVID-19 involving more than 85% of the lungs. It also contains CXR images of healthy lungs without pneumonia or other abnormalities in addition to the COVID-19 data. [50].

### Experimental setup

To study the efficacy of the deep neural network we constructed for the computational evaluation of lung disease severity, we compared its performance to that of other deep learning architectures and examined our ViTReg-IP model against additional datasets. For comparison, many deep-learning approaches were employed. We reveal that the proposed network model is more sensitive and interpretable than the current COVID-Net [40] and COVID-NET-S[34]. We also employ ResNet50, a ResNet variant developed by Kaiming He et al. [52], with 50 layers, where we replace the output layer with a regression head with two outputs. We also tested the Swin transformer [53]. This is a hierarchical transformer architecture whose representation is generated by shifted windows. It can serve as the main structural support for a regression task performed for evaluation. Similarly, the depth-separable regular convolutions of the XceptionNet architecture [54] are put to the test. We also tested the InceptionNet architecture, [55] which emphasizes parallel processing and concurrent feature extraction. Moreover, we tested the model proposed by Cohen et al. [35] which was trained using a large dataset as a feature extractor and allows score predictions. In addition, we tested MobileNetV3, a convolutional neural network tailored to cell phones through a combination of network architecture search (NAS) and the NetAdapt algorithm [56]. This model’s output was also updated to forecast the score using a regression head.

In order to evaluate our experiments, we tested our ViTReg-IP model over several datasets. We trained CXR images of size 224$\times$224 each, 32 images per batch, a learning rate of $1\times 10^-3$, and 60 iterations with *L*1 Loss as the loss function. The Python programming language and the PyTorch Lightning learning package were both employed throughout the whole architecture development phase.

We compute the mean absolute error (MAE) and Pearson correlation coefficient (PC) between the scores predicted by the deep neural networks and the ground-truth scores annotated by expert radiologists for geographic extent, opacity score, Brixia score, and COVID score in the test sub-set of CXR data for each trial in order to measure the performance of the trained models in this study.

### Experimental results and comparison

We used nine different approaches to train the pre-processed and expanded RALO dataset for the assessment of lung severity: COVID-NET [40], COVID-NET-S[34], ResNet50[52], InceptionNet [55], XceptionNet [54], Swin Transformer [53], Stonybrook Feature Extraction [35], MobileNetV3 [56], and our ViTReg-IP model. The dataset contains images labeled with the geographic extent and lung opacity, whose values range from 0 to 8 to denote disease severity, which ranges from normal to severe. The dataset used includes the original images and the images resulting from the previously discussed augmentation methods: offline combined lung and score replacement and online score-correlated CutMix. This applies to all training conducted for all models tested. The models COVID-NET, COVID-NET-S, and Stonybrook Feature Extractor were trained unchanged, while the remaining models were used as a backbone to replace the ViT in our proposed model. The results are shown in Tables 2 and 3. For each metric in each table, a thorough investigation of the performance of deep learning models in assessing infection severity is provided. There are also two columns displaying the number of parameters trained in each model and the duration of training. Table 2 presents the outcomes acquired behind training the models with the geographic extent as a label. It shows that our proposed model has the best performance. Table 3 similarly shows the results for the score of lung opacity. Best results are shown in bold.

**Table 2:** Geographic extent score prediction results

Model	MAE $\downarrow$	PC $\uparrow$	Number of	Training
			parameters	time
COVID-NET[40]	4.563	0.545	12 M	40 min
COVID-NET-S[34]	4.746	0.581	12 M	40 min
ResNet50[52]	1.107	0.684	23 M	1.5 h
Swin Transformer[53]	0.927	0.819	29 M	2 h
XceptionNet [54]	0.864	0.802	23 M	1.5 h
InceptionNet [55]	0.717	0.881	24 M	1.5 h
Feature Extraction[35]	0.981	0.741	20 M	1 h
MobileNetV3[56]	0.864	0.822	4.2 M	40 min
ViTReg-IP (ours)	**0**.**569**	**0**.**923**	**5.5 M**	**20 min**

**Table 3:** Lung opacity score prediction results

Model	MAE $\downarrow$	PC $\uparrow$	Number of	Training
			parameters	time
COVID-NET[40]	2.249	0.531	12 M	40 min
COVID-NET-S[34]	2.227	0.525	12 M	40 min
ResNet50[52]	1.082	0.427	23 M	1.5 h
Swin Transformer[53]	0.811	0.692	29 M	2 h
XceptionNet[54]	0.771	0.696	23 M	1.5 h
InceptionNet[55]	0.614	0.825	24 M	1.5 h
Feature Extraction[35]	0.881	0.701	20 M	1 h
MobileNetV3[56]	0.741	0.731	4.2 M	40 min
ViTReg-IP (ours)	**0**.**512**	**0**.**855**	**5.5 M**	**20 min**

**Table 4:** Results of ViTReg-IP model intra-evaluation

Data	Score	Original	Training	Test	MAE $\downarrow$	PC $\uparrow$
		training size	size*	size		
Brixia	Brixia Score	4695	4695	250	0.981	0.622
Brixia	Brixia Score	4695	9390	250	0.811	0.763
RALO	LO	1878	1878	495	0.881	0.681
RALO	LO	1878	5634	495	0.512	0.855
RALO	GE	1878	1878	495	0.931	0.803
RALO	GE	1878	5634	495	0.596	0.923
Danilov et al. COVID-19	COVID Score	1225	1225	139	0.389	0.951

*if combined lung and score replacement is applied

**Table 5:** Results of ViTReg-IP model cross-evaluation

Training	Test	Score	Original	Training	Test	MAE	PC $\uparrow$
data	data		training size	size*	size		
Brixia	Cohen COVID-19	Brixia Score	4695	4695	192	1.86	0.461
Brixia	Cohen COVID-19	Brixia Score	4695	9390	192	1.23	0.587
RALO	Cohen COVID-19	LO	1878	5634	94	0.857	0.697
RALO	Cohen COVID-19	GE	1878	5634	94	0.838	0.842

*if combined lung and score replacement is applied

**Fig. 5:**
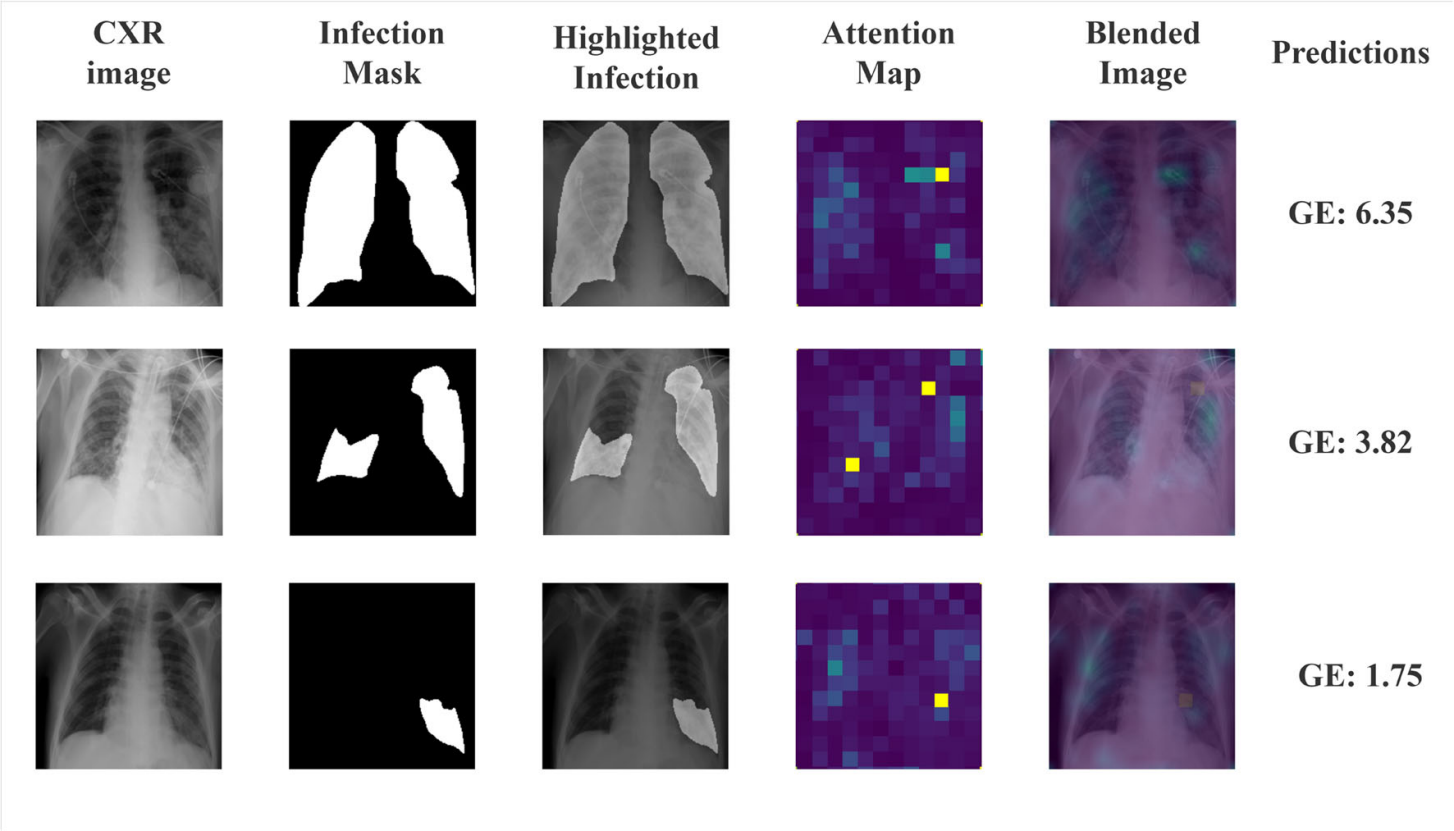
Attention map of CXRs produced by our ViTReg-IP model for GE score. The CXRs are from the QaTa COV19 dataset [57]

To obtain a model with high generalizability, we trained our ViTReg-IP with different combinations of datasets. Depending on the type of data the model was trained with, the results may look different. The experiments included both intra- and cross-evaluation methods. For the intra-evaluation, other than the RALO dataset, the datasets of Brixia and Danilov et al. COVID-19 were used. In each case, the images from the same dataset are divided into training and test data, and the results of the performance of our trained ViTReg-IP model are collected. The data splitting and the results of intra-evaluation are shown in Table 4 to avoid biased performance and to ensure the generalizability of the model, cross-evaluation is tested. Splitting the data into training data from one dataset and test data from another dataset is called cross-evaluation. To avoid any biased performance and confirm the generalizability of the model, cross-evaluation is tested. Several tests were performed by training our ViTReg-IP model with different combinations of datasets. The cross-evaluation results are shown in Table 5. In both intra- and cross-evaluation, experiments are performed on images with and without combined lung and score replacement. This augmentation method can only be applied to data that have separate scores for individual lungs, as in the case of the RALO and Brixia datasets. From Table 5, we can see that the performance of the cross-evaluation was lower than that of the intra-evaluation.

#### Qualitative analysis

We projected the attention maps to demonstrate the effectiveness of our model in identifying areas at risk of infection. Figure 5 shows the ground truth as well as the high-intensity areas corresponding to infection, represented as a feature map. Without the use of sophisticated methods, our recommended model provided a good representation of infection when the ViTReg-IP is trained using geographic extent as a label, with the score correlated to the location of infection. Figure 5 previews some examples of the data collected to evaluate the effectiveness of the proposed method for representing lung infection areas. Since the datasets used in this work do not have a ground-truth mask for infections, we used CXRs from the QaTa COV19 dataset [57]. In Fig. 5, the first column shows the original CXR, the second column shows the actual ground truth of the infection area, the third column shows the image overlaid with the ground truth, column four shows the corresponding attention map, and column five shows a preview of the overlay of the original image with the attention map. The predicted geographic extent values are also included in the last column. The obtained attention maps and predicted scores are highly correlated with the actual location of infection. In addition, the predicted GE scores also correlate with the extent of infection. This indicates that our proposed model has high efficiency in localizing the area of infection concerning the high intensities in the spatial attention map.

**Fig. 6:**
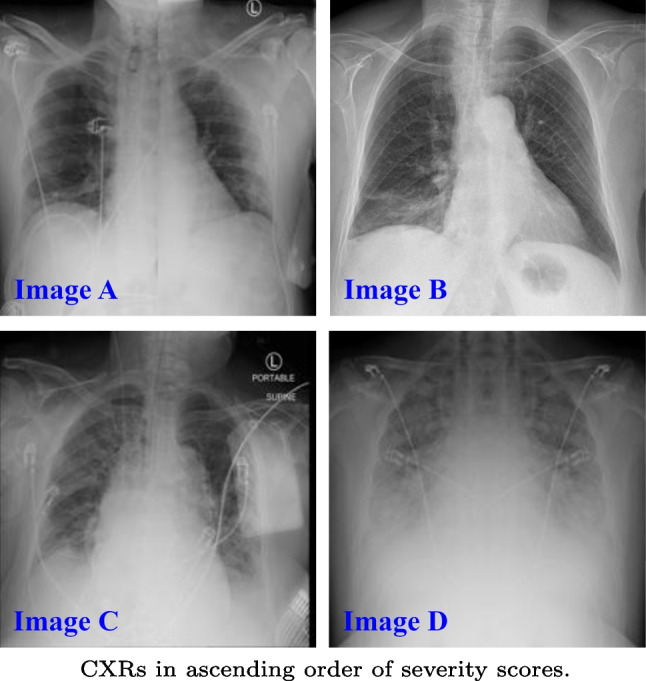
Predictions by the tested models are shown. The CXR images are shown in Fig. 6. The ground truth and predicted scores for both Geographic Extent and Lung Opacity are included in Table 6

**Fig. 7:**
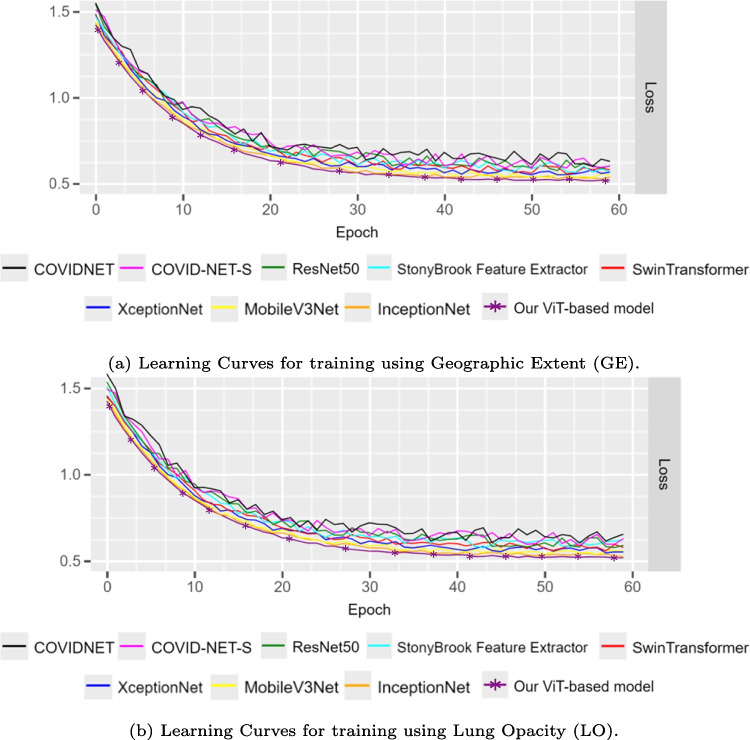
Learning curves for the training of the models

**Fig. 8:**
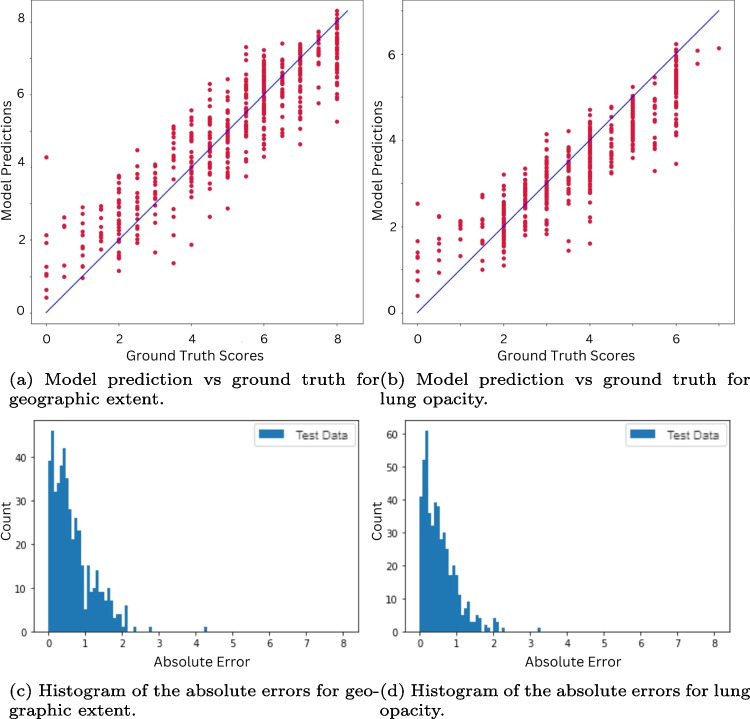
ViTReg-IP evaluations were performed on the test subset

On the other hand, Fig. 6 shows the predictions made for four CXR images using the different deep learning architectures. The images were selected to have different ground-truth scores from the total range to prove that our proposed model is efficient in the whole range of scores. As shown in the table embedded in Fig. 6, the scores expected by our suggested model are most similar to the labeled CXR images as annotated by radiology specialists. Even when there is no infection (scores = 0), as shown in Image A, the estimates for geographic extent and lung opacity are close to zero when compared to the other models. Similarly, the error between the predicted and actual values is smallest when using the ViTReg-IP model in images B, C, and D.

Moreover, Fig. 7 indicates the training performance of the suggested model and the eight *state-of-the-art* models presented over 60 epochs. All of the trained models appear to converge. When the number of epochs increases, the training loss with the proposed model reaches its stable value in the shortest time compared to the other models. The learning curves are shown for both the geographic extent (Fig. 7a) and the lung opacity (Fig. 7b) scores.

**Fig. 9:**
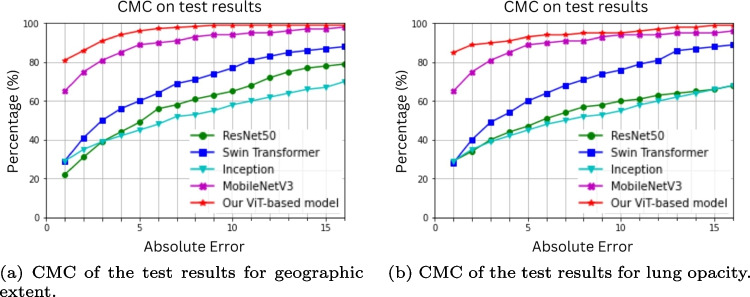
The CMC of a few tested models

**Table 6:** The ground truth and predicted scores for the CXR images provided in Fig. 6

	Prediction							
	Image A		Image B		Image C		Image D	
Model	GE	LO	GE	LO	GE	LO	GE	LO
Ground truth	0	0	1	1.5	6	3	7.5	8
COVID-NET [40]	1.78	1.54	2.01	2.95	2.13	1.52	5.51	4.86
COVID-NET-S [34]	2.01	1.82	2.16	3.12	3.21	2.15	5.13	5.65
ResNet50 [52]	0.97	1.21	1.95	3.14	4.80	2.06	6.92	7.01
Swin Transformer [53]	1.06	0.89	0.567	0.75	4.91	2.54	7.91	6.56
XceptionNet [54]	0.67	1.12	1.57	1.62	5.58	2.52	7.12	6.78
InceptionNet [55]	0.98	0.99	0.53	1.23	6.56	3.51	6.88	6.96
Feature Extraction [35]	1.12	1.01	1.58	0.94	6.84	3.78	7.95	7.15
MobileNetV3 [56]	0.91	0.33	1.61	1.21	5.06	3.29	7.44	7.16
ViTReg-IP (ours)	**0**.**36**	**0**.**27**	**1**.**05**	**1**.**47**	**5**.**48**	**3**.**12**	**7**.**53**	**7**.**96**

#### Quantitative analysis

The quantitative outcomes of the proposed model are shown in Tables 2 and 3. In terms of ground truth versus prediction, our model has obtained the best results. The predicted scores are relatively close to the actual values, as can be seen in Fig. 8 for the RALO dataset. The same is true for both annotations, i.e., geographic extent and lung opacity scores. We also plotted the histograms of the absolute errors obtained with the test images (Fig. 8c and d). From these histograms, we can see that the highest bars are shifted to the left meaning that a large number of test images have a small prediction error. It can be seen that most of the errors of the individual test images are in the range of 0–1, giving the total error.

We also considered the cumulative matching curves (CMC) of some tested models to evaluate their performance. The curves for test images for both scores are shown in Fig. 9. Our proposed model demonstrated superior performance compared to the other four models in the study. Each color represents one model. For the GE score (Fig. 9a), about 80% of the test images in our model have a prediction error below the first error threshold (here it is set to one). The other models tested, such as RestNet50, Swin Transformer, Inception, and MobileNetV3, resulted in a much lower percentage for the same threshold. Similar behavior was obtained with the CMC of the LO score (Fig. 9b).

#### Ablation studies

We performed a series of ablation studies to better understand the contributions of each parameter in our ViTReg-IP model. As shown in Tables, 7, 8, 9, 10, 11, and 12. First, we performed an ablation study to determine the impact of the loss function used on the performance of our suggested model. The model is trained using the CXR images for each loss function, and the results are previewed. The loss functions used are the Huber loss, the MSE loss, and the smooth L1 loss, in addition to the L1 loss. The results are shown in Table 7. The Huber loss function uses a quadratic term to create a criterion if the absolute error is less than a given parameter; otherwise, a scaled L1 term is used. The MSE loss establishes a standard that evaluates the mean squared error between the predicted value and the target value. The smooth L1 loss uses a quadratic term if the absolute error is less than a given parameter and an L1 term otherwise. The results show that using L1 loss as the loss function gives the best results. Using L1 loss, MAE is the smallest with values of 0.569 and 0.512, and PC has the highest values of 0.923 and 0.855 for geographic extent and lung opacity, respectively.

To choose the best optimizer for our model, we trained our ViTReg-IP with five different optimizers and compared the results in terms of MAE and PC. The optimizers tested include Adadelta, SGD, Adam, AdamW, and RMSprop. Table 8 shows the results of the tests and shows that SGD ensures the best performance.

Table 9 shows the ablation study performed for the size of the linear fully connected layer connected to the output of the transformer in the regressor of our ViTReg-IP. We tested a range of sizes and previewed the MAE and PC values corresponding to each test. The results show that the 128 FC layer we chose gives the best results compared to other sizes.

The next study targeted the impact of the augmentation methods on model performance. Training of our ViTReg-IP was performed using either combined lung and score replacement or score-correlated CutMix, both, or neither methods. The results in Table 10 show that combined lung and score replacement (our proposal) made a greater contribution to improving model performance, with MAE decreasing the most and PC increasing the most when applied alone, compared to score-correlated CutMix applied alone. Score-correlated CutMix also improved results but to a lesser extent.

The study revealed in Table 11 consider several online augmentations of the *state-of-the-art*. It was conducted to confirm that choosing CutMix as the online augmentation step produced the best results. The experiments conducted in this concern include several image replacement methods. We used CutOut, which replaces a random box from each image with a black one [58]. Attentive CutMix was also tested where it replaces the most descriptive parts of an image based on the intermediate attention maps of a feature extractor with those of another image [59]. MixUp which performs a fusion of two images to create a new image was also tested [60]. We tested GridMix which uses patch-level label prediction for local context mapping and grid-based mixing [61]. SuperPixelMix uses information merging to create a new style of image augmentation based on superpixel decomposition [62]. PuzzleMix is a MixUp approach that directly uses saliency data and supporting statistics [63]. TransMix is similar to CutMix in terms of mixing images, however, it blends labels based on the Vision Transformers attention matrices [64]. Horizontal image flipping and image blurring are two traditional augmentation methods that were also tested [65]. For all tested augmentation methods that use image mixing, the scoring strategy explained in (5) is applied for each case. As confirmed by the test results in terms of the lowest MAE and the highest PC, the best results were obtained using CutMix as the online data augmentation.

**Table 7:** Ablation study results for loss function performance

	GE		LO	
Loss function	MAE$\downarrow$	PC $\uparrow$	MAE$\downarrow$	PC $\uparrow$
L1Loss	**0**.**569**	**0**.**923**	**0**.**512**	**0**.**855**
MSE Loss	0.590	0.917	0.612	0.817
Smooth L1 Loss	0.615	0.913	0.542	0.843
Huber Loss	0.637	0.909	0.601	0.807

**Table 8:** Ablation study results for optimizer performance

	GE		LO	
Optimizer	MAE $\downarrow$	PC $\uparrow$	MAE $\downarrow$	PC $\uparrow$
SGD	**0**.**569**	**0**.**923**	**0**.**512**	**0**.**855**
Adadelta	0.743	0.881	0.667	0.811
Adam	0.885	0.841	0.939	0.613
AdamW	0.901	0.821	0.813	0.691
RMSprop	1.178	0.697	0.909	0.618

We evaluated several segmentation designs to separate the lung regions from the original CXR image as a preliminary step before training the regression model to see if lung segmentation [66] may improve our model. Consequently, lung segmentation may be thought of as a preprocessing step for the CXR input images. With respect to *state-of-the-art* architectures used for segmenting the lungs from the CXR image, we employed MA-Net [67], PAN [68], and UNet [69]. We decided to test a traditional CNN-based model in addition to our ViTReg-IP. Both models were trained with both scores; the geographic extent and the lung opacity. The results in Table 12 show that similar results are obtained whether or not segmentation is performed before training our proposed model. In addition, we tested ResNet50 as a backbone for the regressor with or without lung segmentation for the CXR input data. The outcomes in terms of MAE and PC are shown in Table 12, which indicates that applying lung segmentation using the MA-Net method improved the performance of the ResNet50-based model.

**Table 9:** Ablation study results for FC layer size performance

	GE		LO	
FC Size	MAE $\downarrow$	PC $\uparrow$	MAE $\downarrow$	PC $\uparrow$
50	0.663	0.922	0.563	0.845
75	0.662	0.921	0.584	0.839
100	0.686	0.910	0.556	0.845
**128**	**0**.**569**	**0**.**923**	**0**.**512**	**0**.**855**
150	0.649	0.901	0.546	0844
175	0.646	0.902	0.529	0.849

**Table 10:** Ablation study results for augmentation performance

Augmentation					
Combined lung	Score-correlated	GE		LO	
and score replacement	CutMix	MAE $\downarrow$	PC $\uparrow$	MAE $\downarrow$	PC $\uparrow$
$\times$	$\times$	1.032	0.778	0.926	0.635
$\checkmark$	$\times$	0.655	0.905	0.573	0.843
$\times$	$\checkmark$	0.931	0.803	0.881	0.681
$\checkmark$	$\checkmark$	**0**.**569**	**0**.**923**	**0**.**512**	**0**.**855**

## Analysis of results and discussions

Compared with the radiologist’s clinical annotations for geographic extent or lung opacity on the entire test set of 495 CXRs, the reported mean absolute errors are less than 0.6, with a range of ground-truth scores of [0, 8]. For a diagnosis of urgency that provides a very accurate assessment of the degree of infection, a MAE of less than 0.6 is considered an acceptable error for the network and radiologists. This phenomenon arises because the ground-truth annotations are discrete values rather than continuous numbers. The scores range from 0 to 8 with increments of 0.5. Consequently, an error less than 0.6 indicates that the predictions are accurately close to the real score, particularly when rounding is applied. Other features of the strategy proposed in this study make it incomparable to similar approaches in the literature. The same experiments are performed with other competing architectures.The experiments are done in which COVID-NET [40], COVID-NET-S [34], and Stonybrook Feature Extraction [35] are trained in addition to training ResNet50 [52], InceptionNet [55], XceptionNet, Swin Transformer [53], and MobileNetV3 [56] as a backbone to the regressor instead of the ViT. The outcomes of training the deep learning architectures are compared with those of our proposed model to demonstrate the value of the work. When compared to other supervised AI-based prediction models, the proposed model outperforms the current deep learning models in terms of MAE and PC, as shown in Tables 2 and 3.

When trained with the processed RALO dataset, the ViTReg-IP model shows commendable performance through empirical validation. The MAE achieved MAE between the predicted values and the radiologist’s scores for both geographic extent (0.596) and opacity extent (0.512) testifies to its remarkable precision. This represents a significant advance and positions the ViTReg-IP model as a leader compared to the current *state-of-the-art*. In addition, the PC measure supports the superiority of the model by recording exceptional values of 0.923 and 0.855 for geographic extent and lung opacity, respectively. These results not only establish the ViTReg-IP model as a state-of-the-art solution but also highlight its unparalleled effectiveness and set a new standard in the field of severity assessment models.

**Table 11:** Ablation study results for online augmentation performance

Online	GE		LO	
augmentation	MAE $\downarrow$	PC $\uparrow$	MAE $\downarrow$	PC $\uparrow$
Score-correlated CutMix	**0**.**569**	**0**.**923**	**0**.**512**	**0**.**855**
TransMix[64]	0.582	0.921	0.551	**0**.**855**
SuperPixelMix[62]	0.789	0.892	0.712	0.873
Horizontal Flip [65]	0.599	0.915	0.574	0.844
Blur [65]	0.602	0.919	0.547	0.843
MixUp[60]	0.611	0.904	0.651	0.837
CutOut[58]	0.642	0.914	0.601	0.814
Attentive CutMix[59]	0.832	0.889	0.789	0.795
PuzzleMix[63]	0.754	0.851	0.721	0.732
GridMix[61]	0.848	0.832	0.834	0.701

**Table 12:** Ablation study results for lung segmentation performance

	ViT-Reg-IP (ours)				ResNet50			
Segmentation	GE		LO		GE		LO	
	MAE $\downarrow$	PC $\uparrow$	MAE $\downarrow$	PC $\uparrow$	MAE $\downarrow$	PC $\uparrow$	MAE $\downarrow$	PC $\uparrow$
No segmentation	**0**.**569**	**0**.**923**	**0**.**512**	**0**.**855**	1.107	0.684	1.082	0.427
Segmentation: MA-Net [67]	0.578	0.919	0.534	0.841	**0**.**798**	**0**.**776**	**0**.**764**	**0**.**762**
Segmentation: PAN [68]	0.589	0.914	0.554	0.831	0.812	0.754	0.809	0.759
Segmentation: UNet [69]	0.654	0.849	0.612	0.798	0.952	0.521	0.935	0.507

When choosing configuration values, considering training costs is as important as focusing on the absolute best performance. For this reason, it is important to examine the model cost, represented by the number of parameters and the training time for each model. This insight is taken into account when selecting a model over a time-consuming training process. Tables 2 and 3 illustrate the computational efficiency indicated by the number of parameters and training time of the proposed model. The proposed model provides the lowest MAE, although it has only 5.5 million parameters that take at most 20 min to train, resulting in a low computational cost.

In addition, Tables 4 and 5 show the generalization and robustness of our model over different combinations of CXR images with different labeling scores. These tests include both intra- and cross-validation tests with different combinations of CXR images.

The ablation studies performed have highlighted the different contributions of multiple parameters. Various options were tested in detail when selecting the loss function, the model optimizer, and the dimensions of the regression head. As described in Section 4.3.3, optimal performance was achieved using the loss function $L_{1}$, the SGD optimizer, and a fully connected (FC) layer with a length of 128. These careful considerations and experiments in choosing these elements emphasize their crucial role in achieving the best possible performance for our model.

In addition, data augmentation made a large contribution to improving the performance of our model, with offline combined lung and score replacement augmentation making the largest contribution. This can be seen in Table 10 by testing the performance of the model with and without offline and online augmentation steps. It is evident that the inclusion of the Lung Replacement and CutMix methods played a crucial role in improving the performance of the ViTReg-IP model, which is reflected in the obtained results of low Mean Absolute Error (MAE) and high Pearson Correlation (PC). While the effects of offline augmentation proved to be particularly influential, the online Score-correlated CutMix also contributed significantly to the improvement in results for both scores. This dual augmentation strategy synergistically improved the robustness and accuracy of the model, confirming its efficiency in handling complex image variations and achieving superior predictive performance in both geographic extent and lung opacity assessment.

The decision to use CutMix as the online augmentation step was made after testing various *state-of-the-art* augmentation methods. As it is shown in Table 11, CutMix, TransMix, and Superpixelmix each provide the best results. The online application of each of these three augmentation methods has significantly increased the performance of the proposed model. Each method involves the strategic blending of two images by exchanging different patches with different approaches. This deliberate manipulation in the creation of new images provides a higher degree of diversity during training and ultimately contributes to a significant increase in the overall performance of the model. Using these augmentation techniques serves to enrich the dataset and provides the model with a wider range of scenarios to learn from, improving its ability to generalize and predict accurately.

Lung segmentation plays a crucial role in deep learning. Its importance lies in its ability to isolate the lung from the surrounding anatomical structures and thus enable a more precise and targeted analysis. However, the results of our experiments show a different outcome. We have shown the results of training our proposed ViTReg-IP with and without segmentation of the input CXRs. Table 11 shows that comparable results were obtained regardless of whether the segmentation was performed before training our proposed model. This is not the case when the same is done with the ResNet50, where the performance increases with segmentation. This discrepancy could be due to the global nature of self-attention used by Vision Transformers, which utilizes information from the entire image. In contrast, CNN-based models rely on a convolution that is influenced by neighboring pixels, which explains the different effects of segmentation on their performance.

## Conclusion

In this study, we hypothesized that a generalized transformer-based approach could reliably and rapidly predict the degree of pulmonary infection in patients with COVID-19 by exploiting multi-score datasets of graded CXRs and comparing them with ground-truth scores annotated by radiologists. The experimental outcomes show the efficacy of the suggested methodology using computer-aided severity evaluation of CXR data from COVID-19-positive patients and its potential to be a helpful tool for clinicians and healthcare workers. The impacts of our experiments demonstrated that the suggested model could be reliably trained with a minimal dataset, compared to *state-of-the-art* approaches, and generated outcomes with the most inferior error, which were strongly associated with radiological values. Moreover, this open-access approach provides a biomedical assistance tool that may be useful for automatically alerting to CXRs scored by physicians, which may require double-checking due to an identified high score deviation. In addition to being highly efficient, our model has the advantage of being computationally inexpensive due to its short training time and not requiring segmentation as a preprocessing step. As a result, the ViTReg-IP can be retrained on new data to respond to the same or different pulmonary infections by predicting the relative corresponding score.

The main limitation of the proposed data augmentation arises from the lack of data annotated with individual scores for the left and right lungs separately. It is worth noting that this limitation only affects the data augmentation phase. This limitation prohibits the direct application of the combined offline lung and score replacement technique in the training phase, as it requires the availability of ground-truth scores for each individual lung. In future work, we aim to address this limitation in the augmentation phase by using the infection masks in the two lungs to calculate the individual scores.

The future trajectory of ViTReg-IP holds promise in advancing towards a multi-faceted approach. One avenue of exploration involves extending the capabilities of ViTReg-IP into a multi-task model, enabling it to predict multiple scores concurrently. This extension aims to enhance the versatility of the model, catering to diverse applications with varying prediction requirements. Furthermore, there is an exciting prospect in the development of an application that seamlessly integrates ViTReg-IP for real-time score predictions. Such an application could find utility in clinical settings, providing rapid and efficient assessments of relevant metrics. Additionally, broadening the scope to incorporate CT scans as input data instead of CXRs represents a compelling direction for future research.

## Data Availability

The data used in this study are mentioned in Section 4.1.
